# Associations of Genetic Risk Score with Obesity and Related Traits and the Modifying Effect of Physical Activity in a Chinese Han Population

**DOI:** 10.1371/journal.pone.0091442

**Published:** 2014-03-13

**Authors:** Jingwen Zhu, Ruth J. F. Loos, Ling Lu, Geng Zong, Wei Gan, Xingwang Ye, Liang Sun, Huaixing Li, Xu Lin

**Affiliations:** 1 Key Laboratory of Nutrition and Metabolism, Institute for Nutritional Sciences, Shanghai Institutes for Biological Sciences, Chinese Academy of Sciences and Graduate University of the Chinese Academy of Sciences, Shanghai, People’s Republic of China; 2 The Charles Bronfman Institute for Personalized Medicine, The Mindich Child Health and Development Institute, The Genetics of Obesity and Related Metabolic Traits Program, Department of Preventive Medicine, The Icahn School of Medicine at Mount Sinai, New York, New York, United States of America; Harvard Medical School, United States of America

## Abstract

**Background/Objectives:**

Recent large-scale genome-wide association studies have identified multiple loci robustly associated with BMI, predominantly in European ancestry (EA) populations. However, associations of these loci with obesity and related traits have not been well described in Chinese Hans. This study aimed to investigate whether BMI-associated loci are, individually and collectively, associated with adiposity-related traits and obesity in Chinese Hans and whether these associations are modified by physical activity (PA).

**Subjects/Methods:**

We genotyped 28 BMI-associated single nucleotide polymorphisms (SNPs) in a population-based cohort including 2,894 unrelated Han Chinese. Genetic risk score (GRS), EA and East Asian ancestry (EAA) GRSs were calculated by adding BMI-increasing alleles based on all, EA and EAA identified SNPs, respectively. Interactions of GRS and PA were examined by including the interaction-term in the regression model.

**Results:**

Individually, 26 of 28 SNPs showed directionally consistent effects on BMI, and associations of four loci (*TMEM18*, *PCSK1*, *BDNF* and *MAP2K5*) reached nominal significance (*P*<0.05). The GRS was associated with increased BMI, trunk fat and body fat percentages; and increased risk of obesity and overweight (all *P*<0.05). Effect sizes (0.11 vs. 0.17 kg/m^2^) and explained variance (0.90% vs. 1.45%) of GRS for BMI tended to be lower in Chinese Hans than in Europeans. The EA GRS and EAA GRS were associated with 0.11 and 0.13 kg/m^2^ higher BMI, respectively. In addition, we found that PA attenuated the effect of the GRS on BMI (*P*_interaction_ = 0.022).

**Conclusions:**

Our observations suggest that the combined effect of obesity-susceptibility loci on BMI tended to be lower in Han Chinese than in EA. The overall, EA and EAA GRSs exert similar effects on adiposity traits. Genetic predisposition to increased BMI is attenuated by PA in this population of Han Chinese.

## Introduction

Obesity is a major risk factor for chronic diseases including type 2 diabetes, hypertension, and cardiovascular diseases [1], and results from the interaction between genes and environmental factors [2]. Recent genome-wide association studies (GWAS) have identified more than 50 loci that are convincingly associated with BMI in populations of European ancestry [3]–[7] and four additional loci (*PCSK1*, *CDKAL1*, *KLF9* and *GP2*) in East Asian populations [8]–[9]. However, Asians in general have a higher body fat percentage than Europeans at any given BMI [10], and the transferability of loci identified in European ancestry populations to Chinese populations has not been extensively studied. Although 18 loci identified in European populations (*FTO*, *SEC16B*, *MC4R*, *GIPR*, *RBJ*, *BDNF*, *MAP2K5*, *GNPDA2*, *TFAP2B*, *TMEM18*, *TMEM160*, *TNNI3K*, *MTCH2*, *FAIM2*, *FLJ35779*, *SH2B1*, *RPL27A* and *NEGR1*) have been confirmed in East Asian populations [9], only 12 BMI-associated single nucleotide polymorphisms (SNPs) identified before 2010 have been investigated for their effects on BMI in Chinese populations [11]–[16]. Furthermore, the collective contribution of these genetic loci to adiposity-related traits has not been studied in Chinese. Therefore, we aimed to examine whether individual and combined SNP effects, evaluated by genetic risk score (GRS), are associated with BMI and adiposity-related traits in Chinese Hans, and whether there are differences in genetic predisposition between Chinese Hans and European ancestry populations. Previous studies have shown that increased physical activity (PA) can attenuate the genetic predisposition to increased BMI and obesity risk [17]–[26]. Therefore, we also examined whether there were interactions between PA and GRS on BMI in Chinese Hans.

## Materials and Methods

### Study Population

The Nutrition and Health of Aging Population in China (NHAPC) study is a population-based study of 3,210 unrelated Chinese Hans, aged 50–70 years living in Beijing or Shanghai. A total of 2,894 individuals had DNA samples are available for genotyping. The study have been described in detail before [27]. A complete physical examination was conducted in local health stations or community clinics, during which body weight and height were measured, and overnight fasting blood sample were collected. PA was assessed by a standardized questionnaire (International Physical Activity Questionnaire, short last 7-day format). Physical activity level for each individual was classified as low, moderate, and high according to the questionnaire protocol (http://www.ipaq.ki.se/scoring.pdf). BMI was calculated as weight/height^2^ (kg/m^2^). Normal weight, overweight and obesity were defined as BMI<24, 24≤BMI<28 and BMI≥28 kg/m^2^, respectively, using the BMI cut-off points for Chinese populations [28]. In addition, 1,016 Shanghai participants completed whole-body DXA scan by Hologic QDR 4500 W scanner (Hologic, Bedford, MA). Total body fat, trunk fat and leg fat, derived using the software provided by the manufacturer, were divided by total body mass to generate their respective percentages in whole body. The phenotypic characteristics of the population are shown in Table S1. The study protocol was approved by the Institutional Review Board of the Shanghai Institute for Nutritional Sciences, and all participants provided written informed consents.

### SNP Selection

Of more than 60 obesity-related SNPs established to date, we focused on the 36 loci identified in GWAS for BMI in European and East Asian ancestry populations [3]–[9]. We selected one representative SNP for each locus. Since eight SNPs were monomorphic in Chinese Hans, a total of 28 BMI-associated SNPs were included in the current analyses, of which 24 SNPs were first identified in European ancestry populations and four SNPs were first identified in East Asian populations.

### Genotyping

Genotyping and imputation of SNPs have been described in detail before [29]. In brief, all samples were genotyped using the Illumina Human660W-Quad BeadChip (Illumina, Inc., San Diego, CA) and sample call rates exceeded 97%. Each SNP had a call rate ≥95% and genotype distribution was in Hardy-Weinberg equilibrium *P*≥0.01. We used the IMPUTE software to impute SNPs from the phase 2 HapMap CHB+JPT (release number 22) reference panel (http://mathgen.stats.ox.ac.uk/impute/impute.html, version 2.1.2), and the 11 imputed SNPs had a high quality (Proper_info>0.8) (Table S2).

### Genotype Risk Score Calculation

Genotypes were coded as 0, 1, or 2 according to the number of BMI-increasing alleles of each SNP, and BMI-increasing alleles were defined according to findings of previous GWAS [3]–[9]. The overall GRS was calculated by summing BMI-increasing alleles of all 28 SNPs. In addition, we calculated a EA GRS by adding the BMI-increasing alleles of the 24 SNPs that reached genome-wide significance (*P*<5×10^−8^) in European ancestry populations [7], and a EAA GRS by adding the 11 BMI-associated SNPs that reached genome-wide significance reported by Wen *et al* and Okada *et al* [8]–[9]. Seven SNPs are shared by EA and EAA GRS (Table S2). Missing genotypes were replaced by the average allele count of respective SNP to calculate GRS. No individuals had more than three missing genotypes.

### Statistical Analysis

We used general linear regression models to test the associations of individual BMI-related SNPs (both the genotyped and imputed) and GRSs with adiposity-related traits, including BMI, body fat percentage, trunk fat percentage and leg fat percentage, assuming an additive effect of the BMI-increasing alleles. In addition, we inverse-normally transformed all outcome variables (to a mean of 0, and SD of 1) and performed the same analyses. This allows us to compare per-allele effect sizes of individual SNPs and of the GRSs across BMI, body fat percentage, trunk fat percentage and leg fat percentage. Logistic regression models were used to test associations between individual SNPs or GRSs and risks of obesity and overweight. Genotype distributions of European ancestry and Chinese Hans were compared using a likelihood ratio test. Heterogeneity of effect sizes between Chinese and Europeans was calculated with Cochran’s Q-test. The explained variance for BMI was calculated as 2*f*(1–f)*β^2^, where f is the minor allele frequency and β is the per-allele effect on standardized values of BMI. Interactions between GRS and PA were tested by including GRS, PA, and the interaction terms (GRS*PA) in general linear regression model. The associations of genetic variants with BMI and obesity and interaction analyses were adjusted for age, age^2^, sex, region (Beijing and Shanghai), and the first two principle components. The associations of genetic variants with body, trunk and leg fat percentage were adjusted for age, age^2^, and sex. The power calculations for association analyses were performed using Quanto software (available at http://hydra.usc.edu/gxe/) using the minor allele frequencies and mean values of BMI from the NHAPC study and the effect sizes originally reported by previous studies [3]–[9]. The power calculations for interaction analyses were performed using code-generating program MLPowSim to generate R code for simulations in interaction analyses [30]. All tests were two-sided, with nominal significance defined as *P*≤0.05. Bonferroni correction was used to adjust for multiple testing in the obesity related traits analyses, and *P*≤0.0004 was considered to be statistical significance. Statistical analyses were performed in R software (version 2.13.1; www.r-project.org).

## Results

Associations with BMI for 26 of the 28 SNPs (binomial test: *P* = 1.52×10^−6^ ) were directionally consistent with those previously reported in Europeans and East Asians, of which four, in or near *TMEM18*, *PCSK1*, *BDNF* and *MAP2K5*, reached nominally significance (*P*<0.05) (Table 1). Two loci, *TFAP2B* identified in Europeans and *KLF9* identified in East Asians only, showed (non-significant) opposite effects on BMI compared to those reported previously. The BMI-increasing alleles were nominally associated with higher body fat percentage for one SNP in *MTIF3* (binomial *P* = 0.002), with greater trunk fat percentage for two SNPs in/near *MTIF3* and *SH2B1* (binomial *P* = 0.006) and with less leg fat percentage for one SNP near *PCSK1* in 28 SNPs (binomial *P* = 0.092) (Table 2). Three BMI-increasing alleles of SNPs in or near *TMEM18* and *PCSK1* were nominally associated with increased risk of obesity (OR was 1.45 and 1.23, *P* = 0.002 on a binomial test) (Table 3). No associations of individual SNPs with adiposity related traits or obesity reached statistical significance (all *P* values>0.004).

**Table 1:** Associations of individual SNPs and GRSs with BMI.

Gene	SNP	EAF	BMI (kg/m^2^)		Explain variance (%)	Power
			Beta (SE)	*P*		
*NEGR1*	rs2568958	0.91	0.02 (0.16)	0.90	0.00	0.20
*TNNI3K*	rs1514175	0.78	0.10 (0.11)	0.38	0.03	0.15
*PTBP2*	rs1555543	0.88	0.14 (0.14)	0.30	0.02	0.11
*SEC16B*	rs574367	0.20	0.19 (0.12)	0.11	0.12	0.59
*TMEM18*	rs11127485	0.91	0.43 (0.16)	0.0083	0.20	0.59
*RBJ*	rs6545814	0.42	0.10 (0.09)	0.30	0.03	0.43
*ETV5*	rs7647305	0.95	0.35 (0.20)	0.087	0.09	0.16
*GNPDA2*	rs10938397	0.30	0.10 (0.10)	0.33	0.03	0.54
*FLJ35779*	rs2112347	0.43	0.06 (0.10)	0.52	0.01	0.27
*PCSK1*	rs261967	0.42	0.23 (0.09)	0.014	0.21	0.81
*CDKAL1*	rs9356744	0.40	0.13 (0.09)	0.16	0.07	0.38
*NUDT3*	rs206936	0.53	0.12 (0.09)	0.18	0.04	0.16
*TFAP2B*	rs987237	0.16	−0.14 (0.12)	0.28	0.04	0.34
*LRRN6C*	rs10968576	0.22	0.11 (0.11)	0.31	0.05	0.25
*KLF9*	rs11142387	0.33	−0.005 (0.10)	0.96	0.00	0.09
*RPL27A*	rs4929949	0.41	0.08 (0.09)	0.37	0.02	0.15
*BDNF*	rs10501087	0.53	0.18 (0.09)	0.050	0.15	0.64
*MTCH2*	rs3817334	0.32	0.07 (0.10)	0.46	0.03	0.15
*FAIM2*	rs7138803	0.28	0.17 (0.10)	0.10	0.09	0.31
*MTIF3*	rs4771122	0.19	0.23 (0.13)	0.067	0.09	0.18
*MAP2K5*	rs4776970	0.24	0.21 (0.11)	0.048	0.12	0.32
*GP2*	rs12597579	0.29	0.04 (0.10)	0.67	0.00	0.10
*SH2B1*	rs4788102	0.14	0.07 (0.13)	0.61	0.00	0.29
*FTO*	rs9939609	0.11	0.12 (0.14)	0.40	0.03	0.83
*MC4R*	rs17782313	0.23	0.01 (0.11)	0.95	0.00	0.66
*KCTD15*	rs29941	0.24	0.05 (0.11)	0.65	0.01	0.13
*GIPR*	rs11671664	0.54	0.04 (0.09)	0.67	0.00	0.47
*TMEM160*	rs3810291	0.29	0.19 (0.10)	0.074	0.12	0.22
GRS		23.4±3.2	0.11 (0.02)	1.54E-07	0.90	0.99
EA GRS		19.2±2.9	0.11 (0.02)	2.11E-06	0.76	0.99
EAA GRS		8.4±2.1	0.13 (0.03)	6.79E-05	0.56	0.99

Data are beta (SE) per BMI-increasing allele, adjusted for age, age^2^, sex, region and the first two principle components. EAF: effect allele frequency.

**Table 2:** Associations of individual SNPs and GRSs with body fat percentage, trunk fat percentage and leg fat percentage.

Gene	SNP	Body fat percentage (%)		Trunk fat percentage (%)		Leg fat percentage (%)	
		Beta (SE)	*P*	Beta (SE)	*P*	Beta (SE)	*P*
*NEGR1*	rs2568958	−0.46 (0.44)	0.30	−0.21 (0.32)	0.50	−0.19 (0.15)	0.19
*TNNI3K*	rs1514175	0.01 (0.29)	0.96	−0.01 (0.21)	0.94	−0.02 (0.10)	0.80
*PTBP2*	rs1555543	0.39 (0.38)	0.30	0.27 (0.27)	0.31	0.09 (0.12)	0.47
*SEC16B*	rs574367	0.003 (0.30)	0.99	0.04 (0.22)	0.84	−0.05 (0.10)	0.59
*TMEM18*	rs11127485	0.79 (0.44)	0.070	0.51 (0.32)	0.11	0.16 (0.14)	0.25
*RBJ*	rs6545814	0.37 (0.25)	0.14	0.28 (0.18)	0.12	0.05 (0.08)	0.53
*ETV5*	rs7647305	0.34 (0.52)	0.52	0.23 (0.38)	0.54	0.08 (0.17)	0.62
*GNPDA2*	rs10938397	0.40 (0.26)	0.13	0.33 (0.19)	0.082	0.02 (0.09)	0.84
*FLJ35779*	rs2112347	−0.18 (0.25)	0.48	−0.18 (0.18)	0.32	0.01 (0.08)	0.90
*PCSK1*	rs261967	−0.35 (0.24)	0.15	−0.16 (0.18)	0.36	−0.16 (0.08)	0.044
*CDKAL1*	rs9356744	0.008 (0.24)	0.97	0.01 (0.17)	0.95	0.04 (0.08)	0.60
*NUDT3*	rs206936	0.09 (0.24)	0.72	−0.02 (0.17)	0.91	0.08 (0.08)	0.31
*TFAP2B*	rs987237	0.56 (0.33)	0.097	0.45 (0.24)	0.065	0.09 (0.11)	0.42
*LRRN6C*	rs10968576	0.38 (0.28)	0.18	0.19 (0.20)	0.34	0.14 (0.09)	0.14
*KLF9*	rs11142387	−0.04 (0.26)	0.87	0.02 (0.19)	0.90	−0.04 (0.09)	0.66
*RPL27A*	rs4929949	0.35 (0.25)	0.16	0.27 ((0.18)	0.13	0.03 (0.08)	0.71
*BDNF*	rs10501087	0.28 (0.24)	0.25	0.21 (0.17)	0.23	0.07 (0.08)	0.39
*MTCH2*	rs3817334	0.28 (0.26)	0.28	0.12 (0.19)	0.51	0.12 (0.09)	0.17
*FAIM2*	rs7138803	0.45 (0.27)	0.094	0.30 (0.19)	0.12	0.12 (0.09)	0.18
*MTIF3*	rs4771122	0.98 (0.34)	0.0047	0.61 (0.25)	0.015	0.21 (0.11)	0.061
*MAP2K5*	rs4776970	0.48 (0.28)	0.085	0.37 (0.20)	0.069	0.05 (0.09)	0.61
*GP2*	rs12597579	0.24 (0.27)	0.36	0.27 (0.19)	0.17	−0.06 (0.09)	0.53
*SH2B1*	rs4788102	0.63 (0.36)	0.077	0.55 (0.26)	0.033	−0.01 (0.12)	0.97
*FTO*	rs9939609	0.26 (0.38)	0.49	0.10 (0.27)	0.72	0.13 (0.12)	0.29
*MC4R*	rs17782313	−0.21 (0.30)	0.48	−0.07 (0.21)	0.74	−0.10 (0.10)	0.31
*KCTD15*	rs29941	0.14 (0.28)	0.61	0.21 (0.20)	0.30	−0.05 (0.09)	0.57
*GIPR*	rs11671664	−0.33 (0.24)	0.17	−0.29 (0.17)	0.088	−0.03 (0.08)	0.68
*TMEM160*	rs3810291	0.13 (0.27)	0.63	0.07 (0.20)	0.74	0.04 (0.09)	0.68
GRS		0.14 (0.05)	0.0090	0.10 (0.04)	0.0082	0.03 (0.02)	0.15
EA GRS		0.18 (0.06)	0.0023	0.12 (0.04)	0.0056	0.04 (0.02)	0.028
EAA GRS		0.08 (0.08)	0.32	0.07 (0.06)	0.21	0.002 (0.03)	0.94

Data are beta (SE) per BMI-increasing allele, adjusted for age, age^2^ and sex.

**Table 3:** Associations of individual SNPs and GRSs with risk of obesity and overweight.

Gene	SNP	Obesity vs. Normal			Overweight vs. Normal		
		OR (95% CI)	*P*	Power	OR (95% CI)	*P*	Power
*NEGR1*	rs2568958	1.09 (0.82–1.45)	0.57	0.14	0.98 (0.82–1.19)	0.87	0.10
*TNNI3K*	rs1514175	0.99 (0.81–1.21)	0.96	0.15	0.93 (0.82–1.06)	0.30	0.16
*PTBP2*	rs1555543	1.05 (0.82–1.347)	0.69	0.06	1.06 (0.90–1.24)	0.51	0.06
*SEC16B*	rs574367	1.13 (0.93–1.37)	0.22	0.50	1.06 (0.93–1.21)	0.41	0.32
*TMEM18*	rs11127485	1.45 (1.07–1.97)	0.016	0.45	1.11 (0.92–1.35)	0.26	0.41
*RBJ*	rs6545814	1.09 (0.92–1.28)	0.31	0.40	1.07 (0.96–1.19)	0.23	0.27
*ETV5*	rs7647305	1.18 (0.82–1.71)	0.37	0.11	1.10 (0.87–1.39)	0.44	0.06
*GNPDA2*	rs10938397	1.09 (0.91–1.30)	0.36	0.41	1.11 (0.99–1.25)	0.080	0.31
*FLJ35779*	rs2112347	0.97 (0.82–1.14)	0.69	0.26	1.01 (0.90–1.13)	0.88	0.19
*PCSK1*	rs261967	1.23 (1.05–1.44)	0.011	0.24	1.10 (0.98–1.22)	0.095	NA
*CDKAL1*	rs9356744	1.11 (0.94–1.31)	0.21	0.68	1.04 (0.93–1.15)	0.53	NA
*NUDT3*	rs206936	1.05 (0.89–1.23)	0.55	0.13	0.98 (0.88–1.09)	0.71	0.15
*TFAP2B*	rs987237	1.05 (0.85–1.29)	0.65	0.35	1.07 (0.93–1.23)	0.38	0.16
*LRRN6C*	rs10968576	1.09 (0.91–1.31)	0.36	0.14	1.07 (0.95–1.22)	0.28	0.12
*KLF9*	rs11142387	0.95 (0.80–1.13)	0.60	0.65	1.06 (0.94–1.18)	0.35	NA
*RPL27A*	rs4929949	1.06 (0.90–1.25)	0.48	0.10	0.97 (0.87–1.08)	0.61	0.06
*BDNF*	rs10501087	1.15 (0.97–1.35)	0.10	0.50	1.11 (0.99–1.23)	0.067	0.32
*MTCH2*	rs3817334	1.01 (0.85–1.20)	0.88	0.07	1.02 (0.91–1.14)	0.79	0.07
*FAIM2*	rs7138803	1.10 (0.93–1.32)	0.27	0.35	1.07 (0.95–1.20)	0.29	0.18
*MTIF3*	rs4771122	1.09 (0.89–1.35)	0.41	0.15	1.06 (0.91–1.22)	0.45	0.10
*MAP2K5*	rs4776970	1.15 (0.95–1.38)	0.14	0.29	1.06 (0.93–1.20)	0.40	0.17
*GP2*	rs12597579	1.07 (0.89–1.27)	0.47	0.28	1.07 (0.95–1.20)	0.26	NA
*SH2B1*	rs4788102	1.06 (0.85–1.31)	0.62	0.14	0.94 (0.81–1.09)	0.38	0.12
*FTO*	rs9939609	1.01 (0.79–1.29)	0.93	0.87	0.99 (0.84–1.16)	0.87	0.58
*MC4R*	rs17782313	1.03 (0.86–1.25)	0.72	0.62	1.03 (0.91–1.17)	0.61	0.53
*KCTD15*	rs29941	0.90 (0.75–1.09)	0.30	0.07	1.06 (0.94–1.20)	0.37	0.08
*GIPR*	rs11671664	0.98(0.84–1.15)	0.80	0.57	0.99 (0.89–1.10)	0.84	0.30
*TMEM160*	rs3810291	1.15 (0.96–1.38)	0.12	0.25	1.04 (0.92–1.17)	0.54	0.09
GRS		1.06 (1.03–1.10)	0.0006	0.99	1.04 (1.01–1.06)	0.0017	0.99
EA GRS		1.06 (1.02–1.10)	0.0044	0.99	1.03 (1.01–1.06)	0.015	0.99
EAA GRS		1.10 (1.04–1.16)	0.0009	0.99	1.06 (1.02–1.09)	0.0038	0.99

Data are OR (95% CI) adjusted for sex, age, age^2^, region and the first two principle components.

**Figure 1:**
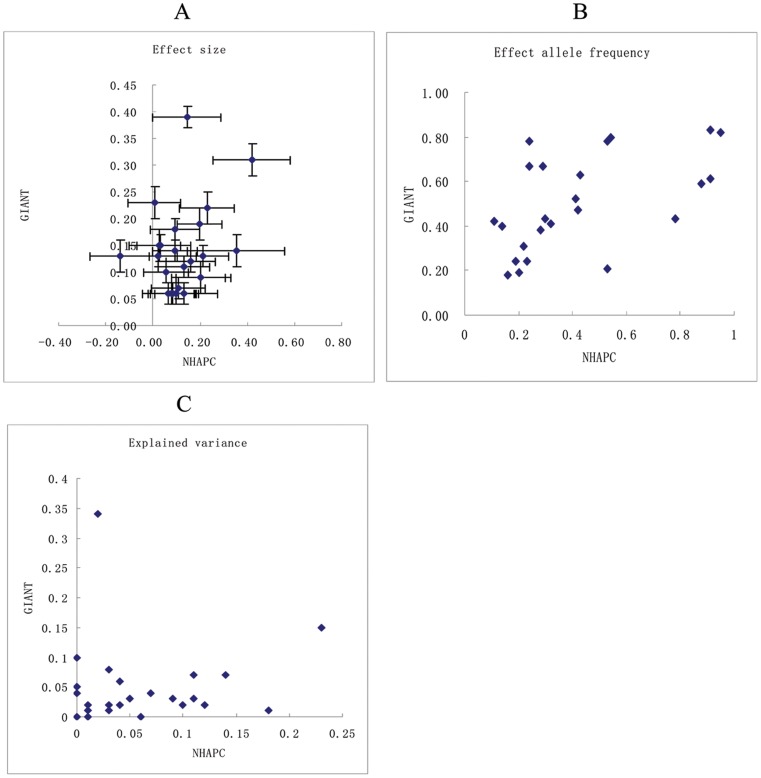
Correlation of BMI-related loci between European ancestry and Chinese Hans. The plots showed the effect size (kg/m^2^) of loci on BMI (A), BMI-increasing allele frequencies (B), and explained variance (%) of loci on BMI (C) in GIANT (Speliotes et al) and NHAPC (our data) studies. X-axis represents the data from our study (NHAPC) and Y-axis represents the data from European ancestry populations (GIANT).

Compared to the effect sizes for the BMI-loci reported in European-ancestry populations by Speliotes *et al* [7], we observed significant inter-ancestry heterogeneity for the effect of *TFAP2B* (*P*_heterogeneity_ *<*0.05), with the effect being larger in Europeans than in Chinese (Table 1, Figure 1A). The BMI-increasing allele frequencies of six SNPs were significantly higher and those of another 19 SNPs were lower in Chinese Hans than in European ancestry populations (*P* for heterogeneity≤0.006), whereas for three SNPs, effect allele frequencies were similar (Table 1, Figure 1B). The explained variances of the four loci that showed nominally significant association with BMI (*TMEM18*, *PCSK1*, *BDNF* and *MAP2K5*) were larger (0.12–0.21% versus 0.01–0.15%) in Chinese than in Europeans (Table 1, Figure 1C).

The GRS, which assesses the combined effect of the 28 SNPs, showed significant association with adiposity-related traits. Each additional BMI-increasing allele in the GRS was associated with 0.11 kg/m^2^ higher BMI (*P* = 1.54×10^−7^; equivalent to 318 g/allele for a person of 1.7 m) (Table 1), 0.14% higher body fat percentage (*P* = 0.0090), and 0.10% higher trunk fat percentage (*P* = 0.0082), but not with leg fat percentage (*P* = 0.15) (Table 2). The effect and explained variance of GRS on BMI tended to be smaller in Chinese (0.11 kg/m^2^; 0.90%) than that in European ancestry (0.17 kg/m^2^; 1.45%).

Using inverse normally transformed BMI-related traits, we found that the GRS of 28 SNPs showed the strongest association with BMI, followed by trunk fat and overall body fat percentage, whereas no association was observed with leg fat percentage (Table S3). The GRS was associated with increased risk of obesity (OR 1.06, 95% CI 1.03–1.10; *P* = 0.0006), and overweight (OR 1.04, 95% CI 1.01–1.06; *P* = 0.0017) (Table 3).

**Figure 2:**
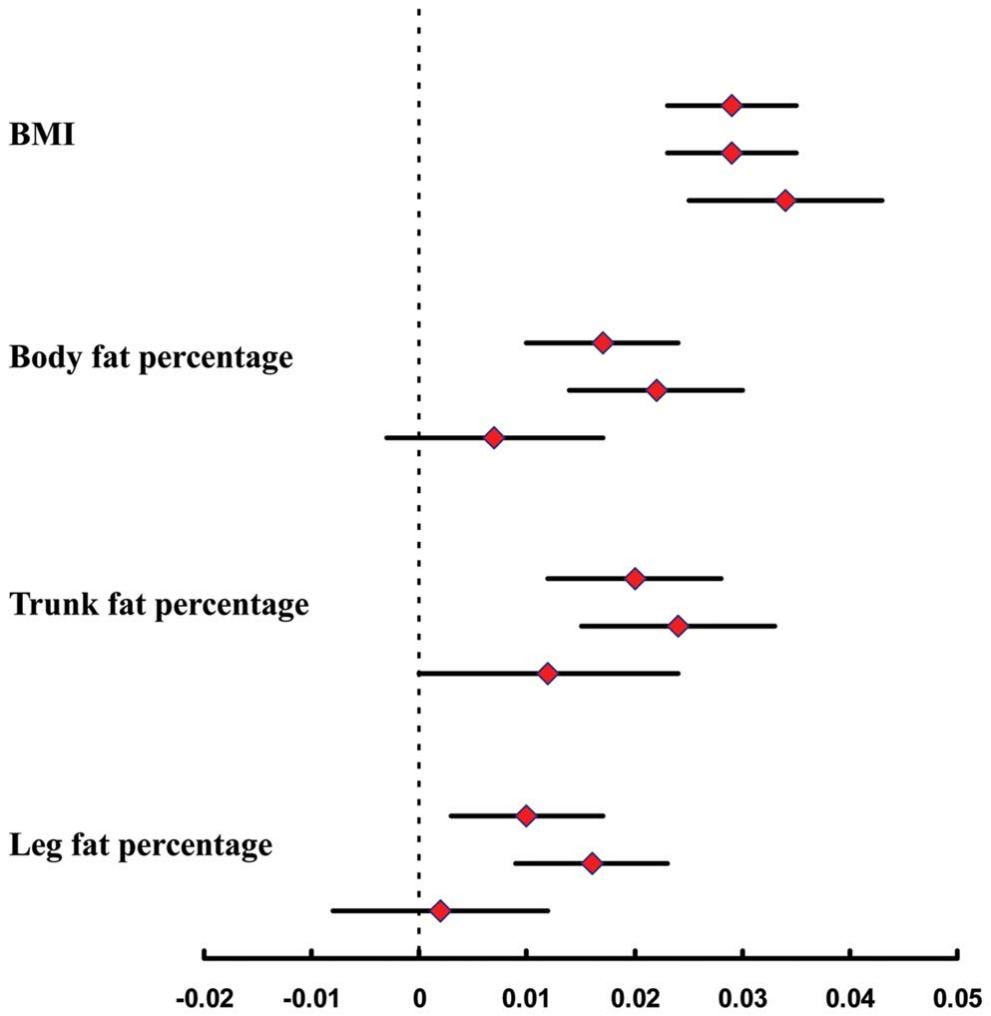
The associations of overall GRS, EA GRS and EAA GRS with adiposity traits. Figure shows the effects of overall GRS (top estimate), EA GRS (middle estimate), and EAA GRS (bottom estimate) on inverse normal transformed BMI, body fat percentage, trunk fat percentage, and leg fat percentage in our population.

Each additional BMI-increasing allele in the European ancestry (EA) GRS, of 24 SNPs, was significantly associated with 0.11 kg/m^2^ higher BMI (*P* = 2.11×10^−6^), whereas the East Asian ancestry (EAA) GRS, of 11 SNPs, was associated with 0.13 kg/m^2^ higher BMI (*P* = 6.79×10^−5^). In addition, both of EA GRS and EAA GRS were associated with increased risk of obesity (OR [95% CI]: 1.06[1.02–1.10]; *P* = 0.0044 and 1.10[1.04–1.16]; *P* = 0.0009, respectively) and overweight (OR [95% CI]: 1.03 (1.01–1.06); *P* = 0.015 and 1.06 (1.02–1.09); *P* = 0.0038, respectively) (Table 3). The EA GRS was significantly associated with body fat distribution including total fat, trunk fat, and leg fat percentage while the associations for EAA GRS did not reach significance (Table 2, Figure 2). However, no heterogeneity was found between the effects of EA and EAA GRS on adiposity traits (*P* for heterogeneity>0.05) (Figure 2).

Increased PA levels significantly attenuated genetic effect of GRS on BMI (beta_interaction_ = −0.06 kg/m^2^, *P*_interaction_ = 0.022; beta_interaction_ represents the difference in the BMI-increasing effect of the GRS on BMI between different levels of PA; Table 4). More specifically, the effect of the GRS on BMI was 60% smaller in people with high physical activity (beta = 0.08 kg/m^2^ per allele; *P* = 0.004) compared to those with low PA (beta = 0.20 kg/m^2^ per allele; *P* = 0.009) (Table 4). The interaction effects were similar for the EA GRS (beta_interaction_ = −0.05 kg/m^2^; *P* = 0.13) and EAA GRS (beta_interaction_ = −0.08 kg/m^2^; *P* = 0.044), but did not reach nominal significance in the former. Individually, *TMEM18*- rs11127485 was nominally significant interacted with PA on BMI (beta_interaction_ = 0.53 kg/m^2^; *P* = 0.04) (Table S4). After *SEC16B*-rs574367 and *FTO*-rs9939609 which were reported interacted with PA on BMI previously [26], and *TMEM18*-rs11127485 were excluded from the GRS, the interaction of combined genetic variants with PA on BMI remained significant (beta_interaction_ = −0.07 kg/m^2^; *P* = 0.04).

**Table 4:** Effect of interaction between GRSs and PA on BMI.

BMI(kg/m^2^)	Main effect of GRS inlow PA	Main effect of GRS inmoderate PA	Main effect of GRSin high PA	GRS×PAInteraction	Power
N	219	1226	1449		
GRS					
Beta(95% CI)	0.20 (0.08)	0.12 (0.03)	0.08 (0.03)	−0.06 (0.03)	0.25
*P*	0.009	0.0003	0.004	0.022	
EA GRS					
Beta(95% CI)	0.15 (0.08)	0.12 (0.04)	0.09 (0.03)	−0.05 (0.04)	0.20
*P*	0.071	0.0008	0.005	0.13	
EAA GRS					
Beta(95% CI)	0.22 (0.12)	0.15 (0.05)	0.09 (0.04)	−0.08 (0.05)	0.37
*P*	0.057	0.003	0.038	0.044	

Interaction beta is the difference in trait of GRS with every increase in PA category, e.g., an interaction beta of −0.06 kg/m^2^ for BMI represents a 0.06 kg/m^2^ attenuation in per BMI-increasing effect of GRS with every increase in PA category. *P*_interaction_ was adjusted for age, age^2^, sex, region and the first two principle components.

## Discussion

In this population-based cohort study of Han Chinese, the majority of the previously identified BMI loci showed directionally consistent effects on BMI, of which four reached nominal significance. The GRS, based on all 28 SNP, was significantly associated with BMI, body fat percentage and trunk fat percentage as well as increased risks of obesity and overweight. Effect size and explained variance of GRS for BMI tended to be lower in Chinese Hans than in European ancestry populations [7]. The overall GRS, EA GRS and EAA GRS had similar effects on adiposity traits. Furthermore, PA attenuated the association between GRS and adiposity related traits; the effect of GRS on BMI was significantly larger in individuals with low compared to those with high physical activity level.

We observed that associations of most loci studied in our Han Chinese population were directionally consistent with those reported for European and East Asian ancestry populations [3]–[9]. Nevertheless, the relative impact of the EA-identified loci in our population tends to be smaller than in European ancestry populations. For example, the *FTO* SNP, which has the largest effect size (0.39 kg/m^2^ per allele) and explained variance (0.34%) for BMI in European ancestry populations [5]–[7], has only a modest effect (0.12 kg/m^2^ per allele; 0.03%) on BMI in our population. Previous finding from meta-analysis conducted in ∼10,000 East and South Asians also provided evidence that the effect of *FTO* on BMI is smaller than that observed in European ancestry [31]. We also found nominally significant ancestry differences in *TFAP2B* that effect size was smaller in Chinese than in EA populations (0.13 versus −0.14 kg/m^2^ per allele) [5]. Even though the effect of *TFAP2B*-rs987237 on BMI was non-significant, the BMI-increasing allele of this SNP was marginally associated with increased trunk fat percentage in our population (*P* = 0.065). This might suggest that *TFAP2B* affects body fat distribution mainly through increasing trunk fat percentage. Although the mechanism of most genetic loci predispose individuals to obesity is not clear so far, evidence indicated that they were involved in energy uptake (*FTO*, *MC4R*, *PCSK1*, and *BDNF*) and adipocyte differentiation (*FTO*, *TMEM18*, *BDNF*, *MTCH2* and *NEGR1*) [32]–[35].

The GRS based on 28 SNPs assesses the overall genetic susceptibility to obesity; each additional risk-allele was associated with a 0.11 kg/m^2^ increase in BMI (equivalent to ∼320 g/allele for a person of 1.7 m), which tended to be smaller than the previously reported 0.17 kg/m^2^ increase of BMI per risk allele in ∼8,000 Europeans based on 32 loci [7]. Another study reported that each additional BMI-increasing allele in the GRS based on 12 SNPs was associated with a 0.15 kg/m^2^ increase of BMI in ∼20,000 Europeans [23]. Consistently, the GRS explained less variance of BMI in our populations (0.90%) than in European ancestry populations (1.45%) [7]. Furthermore, each additional BMI-increasing allele increased the risk of obesity by 6% in the present study but approximately 10% in European ancestry populations [23], [36]. These differences maybe due to the fact that the majority of SNPs (24 of 28) in the score were identified in European ancestry at genome-wide significant levels, whereas only 11 of the SNPs did so in East Asians. Besides, smaller minor allele frequency of 19 SNPs in Chinese population than in Europeans and Winner’s curse effect might be important contributions to the cross-population differences [37]. Nevertheless, the conclusions are expected since it is well established that the SNPs from GWAS have limited predictive ability to distinguish individuals with obesity from ones with normal weight [38]–[39].

We subsequently compared the effect of the GRS across adiposity and body composition traits and observed that GRS showed the strongest association with BMI, followed by trunk fat and overall body fat percentage. This may due to that the GRS is consisted of loci identified in GWAS for BMI rather than for body composition traits. Although BMI is a good marker for general adiposity, it does not distinguish between lean and fat body mass, as well as regional fat distribution.

Contrary to our hypothesis that the EAA GRS, based on East Asian-identified SNPs, would have a larger effect on BMI in our population than the EA GRS, based on European-identified SNPs, the effects of EA GRS on BMI and obesity risk were largely similar to that of EAA GRS. Interestingly, effects of EAA GRS on other adiposity traits tended to be smaller than those of EA GRS. The reason for this discrepancy is not clear. However, we speculate that this might be related to the fact the BMI represents a somewhat different adiposity phenotype in East Asian than in European ancestry populations, such that BMI-associated loci identified in European ancestry populations are, to some extent, different from BMI-associated loci identified in East Asian populations. Although no heterogeneity was found between effects of EA and EAA GRS on adiposity traits, this may attribute to the limited sample size of our current study, which provided a too wide confidence interval to test the significance for effects heterogeneity between EAA GRS and EA GRS. In this regard, larger studies with detailed adiposity phenotypes will be required to confirm these observations.

Consistent with observations in European ancestry populations [17], [20], [23], [26], we found that the effect of GRS on obesity-susceptibility was more pronounced in individuals with low PA than in those with high PA levels. While each additional risk allele increases the BMI by 0.08 kg/m^2^ in physically active individuals, the increase was 0.20 kg/m^2^ per allele in physically inactive individuals, or a difference of 60%, which is twice the difference seen in European ancestry [17], [20], [23], [26]. More mechanism studies on how PA affects the genetic association are needed.

We acknowledge that the sample size in the present study had enough powers (≥99%) for detecting associations of the GRSs with obesity and related traits, but less than 80% power for detecting betas, as previously reported, of most individual SNPs (except for *PCSK1*-rs261967 and *FTO*-rs9939609) for associations with BMI, and for the gene×physical activity interactions in obesity. We, therefore, cannot rule out the possibility of false negative results for these analyses and more study with large sample size is required for making firm conclusion.

In conclusion, the effect size and explained variance of GRS on BMI tends to be lower in Chinese Hans than European ancestry, and the effects of GRSs based on all loci, EA-identified loci or EAA-identified loci, are the same for BMI and obesity risk. Furthermore, the effect of GRS and BMI is attenuated by 60% in physically active individuals.

## Supporting Information

Table S1 Characteristics of the study population.
(DOC)

Table S2 Characteristics of 28 established SNPs.
(DOCX)

Table S3 Associations of individual SNPs and GRS with inverse normally transformed BMI, body fat percentage, trunk fat percentage and leg fat percentage.
(DOCX)

Table S4 Interactions of individual SNPs and physical activity on BMI.
(DOCX)
