# Supplementary material for: Associations of Genetic Risk Score with Obesity and Related Traits and the Modifying Effect of Physical Activity in a Chinese Han Population
Source: PLoS One. 2014 Mar 13;9(3):e91442. doi: 10.1371/journal.pone.0091442 (PMC3953410; doi:10.1371/journal.pone.0091442)
Supplement: Table S1 — Characteristics of the study population. (DOC) [file pone.0091442.s001.doc]

**Table S1** Characteristics of the study population.

|  | Men | Women | *P* | All |
| --- | --- | --- | --- | --- |
| N | 1251 | 1643 |  | 2894 |
| Age (years) | 58.9±5.9 | 58.4±6.1 | 0.53 | 58.6±6.0 |
| BMI (kg/m^2^) | 24.1±3.4 | 24.7±3.8 | 0.00001 | 24.5±3.6 |
| Physical activity level (%) |  |  | 0.54 |  |
| Low | 86 (6.87) | 133 (8.09) |  | 219 (7.57) |
| Moderate | 489 (39.09) | 737 (44.86) |  | 1226 (42.36) |
| High | 676 (54.65) | 773 (47.05) |  | 1449 (50.07) |
| Body fat percentage (%) | 20.4±5.4 | 32.4±5.3 | 0.71 | 27.6±7.9 |
| Trunk fat percentage (%) | 11.4±3.8 | 17.2±3.9 | 0.65 | 14.8±4.8 |
| Leg fat percentage (%) | 5.5±1.4 | 9.7±2.0 | 1.37E-11 | 8.0±2.7 |
| Obesity (%) | 160 (12.8) | 282 (17.2) | 0.002 | 442 (15.3) |
| Overweight (%) | 466 (37.3) | 626 (38.1) | 0.83 | 1092 (37.7) |

Data are means ± SD or N (%) unless otherwise indicated.
