# Supplementary material for: Associations of Genetic Risk Score with Obesity and Related Traits and the Modifying Effect of Physical Activity in a Chinese Han Population
Source: PLoS One. 2014 Mar 13;9(3):e91442. doi: 10.1371/journal.pone.0091442 (PMC3953410; doi:10.1371/journal.pone.0091442)
Supplement: Table S2 — Characteristics of 28 established SNPs. (DOCX) [file pone.0091442.s002.docx]

**Table S2** Characteristics of 28 established SNPs

| Gene | SNP | Chr | Position | Major allele | Minor allele | MAF | HWE  (*p*-value) | Proper_info |
| --- | --- | --- | --- | --- | --- | --- | --- | --- |
| SNPs identified both in European ancestry and East Asians ^1,2,3^ | | | | | | | | |
| *SEC16B* | rs574367 | 1 | 176139833 | G | **T** | 0.20 | 0.36 | 0.99 |
| *RBJ* | rs6545814 | 2 | 24984820 | A | **G** | 0.42 | 0.97 | 1.00 |
| *BDNF* | rs10501087 | 11 | 27636492 | **T** | C | 0.47 | 0.04 | 1.00 |
| *MAP2K5* | rs4776970 | 15 | 65867940 | T | **A** | 0.24 | 0.92 | 0.98 |
| *FTO* | rs9939609 | 16 | 52370868 | T | **A** | 0.11 | 0.23 | 1.00 |
| *MC4R* | rs17782313 | 18 | 55980115 | T | **C** | 0.23 | 0.43 | 1.00 |
| *GIPR* | rs11671664 | 19 | 50864118 | **G** | A | 0.46 | 0.82 | 1.00 |
| SNPs identified in European ancestry only ^1,2^ | | | | | | | | |
| *NEGR1* | rs2568958 | 1 | 72537704 | **A** | G | 0.09 | 0.56 | 1.00 |
| *TNNI3K* | rs1514175 | 1 | 74764232 | **A** | G | 0.22 | 0.01 | 1.00 |
| *PTBP2* | rs1555543 | 1 | 96717385 | **C** | A | 0.12 | 0.93 | 0.99 |
| *TMEM18* | rs11127485 | 2 | 622028 | **T** | C | 0.09 | 0.26 | 0.99 |
| *ETV5* | rs7647305 | 3 | 187316984 | **C** | T | 0.05 | 0.47 | 1.00 |
| *GNPDA2* | rs10938397 | 4 | 44877284 | A | **G** | 0.30 | 0.04 | 1.00 |
| *FLJ35779* | rs2112347 | 5 | 75050998 | G | **T** | 0.43 | 0.03 | 1.00 |
| *NUDT3* | rs206936 | 6 | 34410847 | **G** | A | 0.47 | 0.26 | 0.98 |
| *TFAP2B* | rs987237 | 6 | 51005210 | A | **G** | 0.16 | 0.34 | 1.00 |
| *LRRN6C* | rs10968576 | 9 | 28404339 | A | **G** | 0.22 | 0.08 | 1.00 |
| *RPL27A* | rs4929949 | 11 | 8561169 | T | **C** | 0.41 | 0.42 | 0.95 |
| *MTCH2* | rs3817334 | 11 | 47607569 | C | **T** | 0.32 | 0.44 | 0.99 |
| *FAIM2* | rs7138803 | 12 | 48533735 | G | **A** | 0.28 | 0.61 | 1.00 |
| *MTIF3* | rs4771122 | 13 | 26918180 | A | **G** | 0.19 | 0.39 | 0.93 |
| *SH2B* | rs4788102 | 16 | 28780899 | G | **A** | 0.14 | 0.02 | 1.00 |
| *KCTD15* | rs29941 | 19 | 39001372 | A | **G** | 0.24 | 0.84 | 1.00 |
| *TMEM160* | rs3810291 | 19 | 52260843 | G | **A** | 0.29 | 0.39 | 0.94 |
| SNPs identified in East Asians only ^1,3^ | | | | | | | | |
| *PCSK1* | rs261967 | 5 | 95876006 | A | **C** | 0.42 | 0.85 | 1.00 |
| *CDKAL1* | rs9356744 | 6 | 20793465 | **T** | C | 0.40 | 0.28 | 0.99 |
| *KLF9* | rs11142387 | 9 | 72188152 | A | **C** | 0.33 | 1.00 | 0.97 |
| *GP2* | rs12597579 | 16 | 20165368 | **C** | T | 0.29 | 0.62 | 0.99 |

Chr: chromosome; MAF: Minor allele frequency; HWE: Hardy-Weinberg equilibrium; Proper_info: imputation quality of SNPs.

Allele with underline is BMI-increasing allele.

^1^ SNPs included in the overall GRS; ^2^ SNPs included in the EA GRS; ^3^ SNPs included in the EAA GRS. GRS is the sum of BMI-increasing alleles of 28 SNPs, EA GRS is the sum of BMI-increasing alleles of 24 SNPs identified in European populations, and EAA GRS is the sum of BMI-increasing alleles of 11 SNPs reached genome-wide significance (*P*<5E-08) in East Asian populations.
