# Supplementary material for: Associations of Genetic Risk Score with Obesity and Related Traits and the Modifying Effect of Physical Activity in a Chinese Han Population
Source: PLoS One. 2014 Mar 13;9(3):e91442. doi: 10.1371/journal.pone.0091442 (PMC3953410; doi:10.1371/journal.pone.0091442)
Supplement: Table S3 — Associations of individual SNPs and GRS with inverse normally transformed BMI, body fat percentage, trunk fat percentage and leg fat percentage. (DOCX) [file pone.0091442.s003.docx]

**Table S3** Associations of individual SNPs and GRS with inverse normally transformed BMI, body fat percentage, trunk fat percentage and leg fat percentage.

| Gene | SNP | BMI (kg/m^2^) | |  | Body fat percentage (%) | |  | Trunk fat percentage (%) | |  | Leg fat percentage (%) | |
| --- | --- | --- | --- | --- | --- | --- | --- | --- | --- | --- | --- | --- |
|  |  | Beta (SE) | *P* |  | Beta (SE) | *P* |  | Beta (SE) | *P* |  | Beta (SE) | *P* |
| *NEGR1* | rs2568958 | -0.005 (0.045) | 0.92 |  | -0.058 (0.058) | 0.32 |  | -0.044 (0.067) | 0.51 |  | -0.073 (0.056) | 0.19 |
| *TNNI3K* | rs1514175 | -0.029 (0.032) | 0.36 |  | 0.007 (0.038) | 0.85 |  | -0.006 (0.044) | 0.89 |  | -0.019 (0.037) | 0.60 |
| *PTBP2* | rs1555543 | 0.035 (0.039) | 0.37 |  | 0.047 (0.050) | 0.34 |  | 0.051 (0.057) | 0.37 |  | 0.034 (0.048) | 0.48 |
| *SEC16B* | rs574367 | 0.058 (0.032) | 0.068 |  | -0.008 (0.040) | 0.84 |  | 0.002 (0.046) | 0.96 |  | -0.022 (0.038) | 0.57 |
| *TMEM18* | rs11127485 | 0.115 (0.045) | **0.011** |  | 0.096 (0.058) | 0.094 |  | 0.091 (0.066) | 0.17 |  | 0.062 (0.056) | 0.26 |
| *RBJ* | rs6545814 | 0.027 (0.026) | 0.29 |  | 0.047 (0.033) | 0.16 |  | 0.053 (0.038) | 0.16 |  | 0.023 (0.032) | 0.48 |
| *ETV5* | rs7647305 | 0.095 (0.057) | 0.095 |  | 0.047 (0.069) | 0.49 |  | 0.053 (0.079) | 0.51 |  | 0.041 (0.066) | 0.54 |
| *GNPDA2* | rs10938397 | 0.026 (0.028) | 0.36 |  | 0.052 (0.034) | 0.13 |  | 0.071 (0.040) | 0.073 |  | 0.009 (0.033) | 0.78 |
| *FLJ35779* | rs2112347 | 0.014 (0.026) | 0.61 |  | -0.023 (0.032) | 0.47 |  | -0.041 (0.037) | 0.27 |  | -0.0002 (0.031) | 0.99 |
| *PCSK1* | rs261967 | 0.061 (0.026) | **0.018** |  | -0.041 (0.032) | 0.20 |  | -0.033 (0.037) | 0.37 |  | -0.055 (0.031) | 0.076 |
| *CDKAL1* | rs9356744 | 0.037 (0.026) | 0.15 |  | -0.003 (0.031) | 0.92 |  | -0.0001 (0.036) | 0.99 |  | 0.016 (0.030) | 0.59 |
| *NUDT3* | rs206936 | 0.026 (0.026) | 0.31 |  | 0.021 (0.031) | 0.50 |  | 0.001 (0.036) | 0.98 |  | 0.029 (0.030) | 0.34 |
| *TFAP2B* | rs987237 | -0.044 (0.034) | 0.20 |  | 0.076 (0.044) | 0.086 |  | 0.104 (0.051) | **0.039** |  | 0.024 (0.043) | 0.58 |
| *LRRN6C* | rs10968576 | 0.035 (0.030) | 0.25 |  | 0.044 (0.037) | 0.23 |  | 0.046 (0.042) | 0.28 |  | 0.047 (0.036) | 0.19 |
| *KLF9* | rs11142387 | -0.007 (0.028) | 0.80 |  | -0.004 (0.034) | 0.91 |  | 0.001 (0.039) | 0.97 |  | -0.011 (0.033) | 0.73 |
| *RPL27A* | rs4929949 | 0.026 (0.026) | 0.32 |  | 0.049 (0.032) | 0.13 |  | 0.055 (0.037) | 0.14 |  | 0.015 (0.031) | 0.62 |
| *BDNF* | rs10501087 | 0.054 (0.026) | **0.036** |  | 0.037 (0.032) | 0.25 |  | 0.045 (0.037) | 0.22 |  | 0.026 (0.031) | 0.40 |
| *MTCH2* | rs3817334 | 0.027 (0.028) | 0.32 |  | 0.034 (0.034) | 0.32 |  | 0.033 (0.039) | 0.40 |  | 0.039 (0.033) | 0.24 |
| *FAIM2* | rs7138803 | 0.046 (0.029) | 0.11 |  | 0.058 (0.035) | 0.10 |  | 0.061 (0.041) | 0.14 |  | 0.056 (0.034) | 0.10 |
| *MTIF3* | rs4771122 | 0.059 (0.035) | 0.092 |  | 0.133 (0.045) | **0.0034** |  | 0.117 (0.052) | **0.025** |  | 0.082 (0.044) | 0.062 |
| *MAP2K5* | rs4776970 | 0.056 (0.030) | 0.063 |  | 0.060 (0.037) | 0.11 |  | 0.073 (0.043) | 0.086 |  | 0.015 (0.036) | 0.68 |
| *GP2* | rs12597579 | 0.012 (0.028) | 0.67 |  | 0.026 (0.035) | 0.46 |  | 0.047 (0.040) | 0.25 |  | -0.017 (0.034) | 0.61 |
| *SH2B1* | rs4788102 | 0.014 (0.036) | 0.69 |  | 0.075 (0.047) | 0.11 |  | 0.112 (0.054) | **0.038** |  | -0.003 (0.046) | 0.94 |
| *FTO* | rs9939609 | 0.031 (0.040) | 0.43 |  | 0.031 (0.050) | 0.54 |  | 0.014 (0.057) | 0.81 |  | 0.047 (0.048) | 0.33 |
| *MC4R* | rs17782313 | 0.002 (0.031) | 0.94 |  | -0.031 (0.039) | 0.42 |  | -0.018 (0.045) | 0.68 |  | -0.043 (0.038) | 0.25 |
| *KCTD15* | rs29941 | 0.019 (0.030) | 0.52 |  | 0.023 (0.037) | 0.53 |  | 0.053 (0.042) | 0.21 |  | -0.028 (0.036) | 0.44 |
| *GIPR* | rs11671664 | 0.005 (0.026) | 0.84 |  | -0.048 (0.031) | 0.12 |  | -0.064 (0.036) | 0.076 |  | -0.005 (0.030) | 0.86 |
| *TMEM160* | rs3810291 | 0.056 (0.029) | 0.054 |  | 0.007 (0.036) | 0.85 |  | 0.006 (0.041) | 0.89 |  | 0.014 (0.034) | 0.68 |
| GRS |  | 0.030 (0.006) | 2.20E-07 |  | 0.017 (0.007) | 0.013 |  | 0.020 (0.008) | 0.013 |  | 0.010 (0.007) | 0.12 |
| EA GRS |  | 0.030 (0.006) | 2.19E-06 |  | 0.022 (0.008) | 0.0034 |  | 0.024 (0.009) | 0.0072 |  | 0.016 (0.007) | 0.029 |
| EAA GRS |  | 0.034 (0.009) | 5.77E-05 |  | 0.008 (0.010) | 0.46 |  | 0.012 (0.012) | 0.30 |  | 0.002 (0.010) | 0.87 |

Data are beta (SE) per BMI-increasing allele, adjusted for age, age^2^, sex, region and the first two principle components.
