# Supplementary material for: Associations of Genetic Risk Score with Obesity and Related Traits and the Modifying Effect of Physical Activity in a Chinese Han Population
Source: PLoS One. 2014 Mar 13;9(3):e91442. doi: 10.1371/journal.pone.0091442 (PMC3953410; doi:10.1371/journal.pone.0091442)
Supplement: Table S4 — Interactions of individual SNPs and physical activity on BMI. (DOCX) [file pone.0091442.s004.docx]

**Table S4** Interactions of individual SNPs and physical activity on BMI.

| Gene | SNP | Beta _G*E_ (SE) | *P* | Power |
| --- | --- | --- | --- | --- |
| *NEGR1* | rs2568958 | -0.26 (0.25) | 0.30 | 0.11 |
| *TNNI3K* | rs1514175 | -0.06 (0.18) | 0.75 | 0.06 |
| *PTBP2* | rs1555543 | -0.01 (0.22) | 0.96 | 0.05 |
| *SEC16B* | rs574367 | 0.06 (0.19) | 0.74 | 0.06 |
| *TMEM18* | rs11127485 | 0.53 (0.26) | 0.04 | 0.22 |
| *RBJ* | rs6545814 | 0.27 (0.15) | 0.08 | 0.19 |
| *ETV5* | rs7647305 | -0.40 (0.34) | 0.24 | 0.13 |
| *GNPDA2* | rs10938397 | 0.07 (0.16) | 0.69 | 0.07 |
| *FLJ35779* | rs2112347 | -0.10 (0.15) | 0.52 | 0.09 |
| *PCSK1* | rs261967 | -0.06 (0.15) | 0.70 | 0.07 |
| *CDKAL1* | rs9356744 | -0.25 (0.15) | 0.09 | 0.17 |
| *NUDT3* | rs206936 | -0.09 (0.15) | 0.56 | 0.08 |
| *TFAP2B* | rs987237 | -0.19 (0.20) | 0.33 | 0.11 |
| *LRRN6C* | rs10968576 | -0.28 (0.18) | 0.11 | 0.16 |
| *KLF9* | rs11142387 | -0.10 (0.16) | 0.52 | 0.08 |
| *RPL27A* | rs4929949 | -0.15 (0.15) | 0.33 | 0.11 |
| *BDNF* | rs10501087 | 0.01 (0.15) | 0.93 | 0.05 |
| *MTCH2* | rs3817334 | -0.15 (0.16) | 0.35 | 0.11 |
| *FAIM2* | rs7138803 | -0.22 (0.16) | 0.18 | 0.14 |
| *MTIF3* | rs4771122 | 0.12 (0.21) | 0.57 | 0.08 |
| *MAP2K5* | rs4776970 | -0.32 (0.18) | 0.07 | 0.19 |
| *GP2* | rs12597579 | -0.02 (0.16) | 0.92 | 0.06 |
| *SH2B1* | rs4788102 | -0.05 (0.21) | 0.82 | 0.06 |
| *FTO* | rs9939609 | -0.45 (0.24) | 0.06 | 0.20 |
| *MC4R* | rs17782313 | 0.004 (0.18) | 0.98 | 0.05 |
| *KCTD15* | rs29941 | 0.20 (0.17) | 0.24 | 0.12 |
| *GIPR* | rs11671664 | -0.12 (0.15) | 0.41 | 0.10 |
| *TMEM160* | rs3810291 | 0.06 (0.17) | 0.70 | 0.07 |
| GRS with three SNPs excluded^*^ |  | -0.07 (0.03) | 0.04 | 0.23 |

*GRS excluded *SEC16B*-rs574367, *TMEM18*- rs11127485, and *FTO*-rs9939609.
